# Supplementary material for: The phase separation underlying the pyrenoid-based microalgal Rubisco supercharger
Source: Nat Commun. 2018 Nov 29;9:5076. doi: 10.1038/s41467-018-07624-w (PMC6265248; doi:10.1038/s41467-018-07624-w)
Supplement: Supplementary file 1 — Supplementary Information [file 41467_2018_7624_MOESM1_ESM.pdf]

SUPPLEMENTARY INFORMATION

The phase separation underlying the pyrenoid-based microalgal Rubisco supercharger

Tobias Wunder, Steven Cheng Le Hung, Lai Soak Kuan, Li Hoi Yeung and Oliver Mueller-Cajar*

School of Biological Sciences, Nanyang Technological University, 60 Nanyang Drive, Singapore 637551. Singapore

*Correspondence should be addressed to O. M.-C. email: cajar@ntu.edu.sg

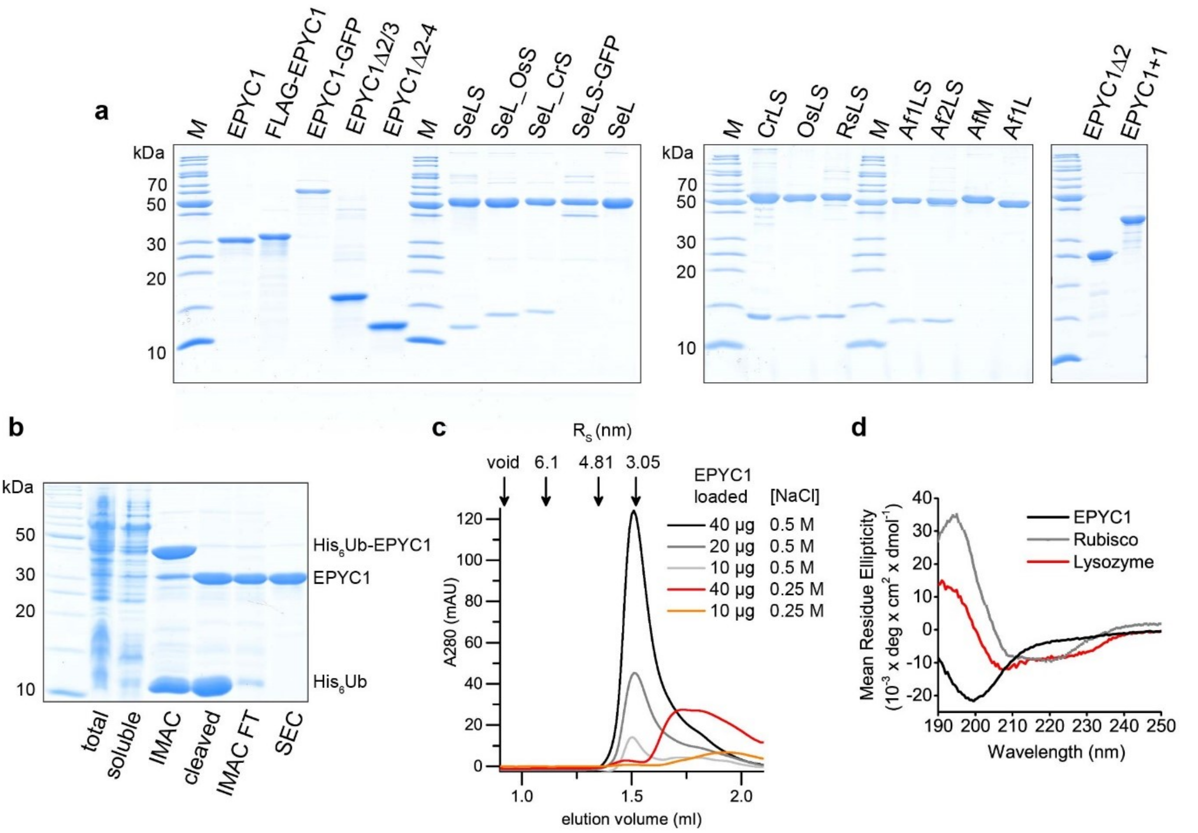

Supplementary Figure 1| SDS-PAGE analysis of proteins used in this study and the purification and properties of EPYC1. a, SDS-PAGE analysis of pure proteins. Rubisco abbreviations: Se, *Synechococcus* PCC6301; Os, rice; Cr, Chlamydomonas; Rs, *Rhodobacter sphaeroides*; Af, *Acidithiobacillus ferrooxidans*; L, large subunit; S, small subunit; M, Form II large subunit. **b,** A representative purification of EPYC1. IMAC, eluted fraction after immobilized metal affinity chromatography; cleaved, IMAC fraction following cleavage of His_6-Ub moiety; IMAC FT, flowthrough of reapplication to IMAC (resulting in His_6-Ub removal); SEC, size exclusion chromatography. **c**, Analytical gel filtration was performed using a Superdex 200 Increase 10/300 column in buffer A. The R_S was obtained by comparison with known standards. At reduced [NaCl] the elution was retarded presumably due to interaction with the resin material. **d**, CD spectroscopy of EPYC1 (0.15 mg/ml, 20 mM phosphate buffer pH 8.0) is consistent with a disordered structure (low ellipticity above 210 nm and negative bands near 195 nm). Well folded proteins (CrRubisco and Lysozyme) were measured in parallel as controls.

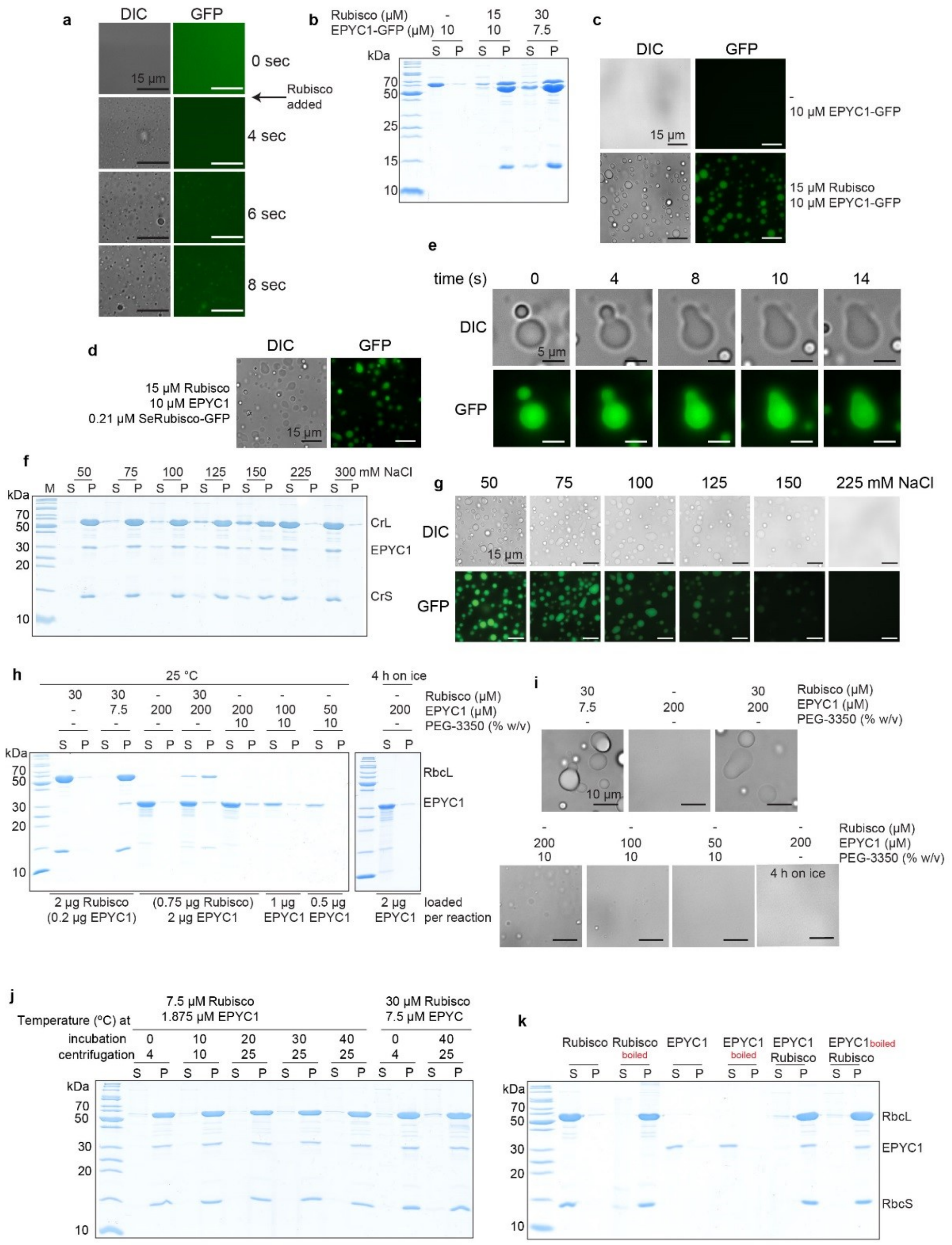

Supplementary Figure 2| Characterization of the Rubisco-EPYC1 phase separation. a, Time lapse imaging of droplet formation following Rubisco (30 µM) addition to EPYC1/EPYC1-GFP (7.1/0.4 µM). Scale bar 15 µm. **b,c** Droplet sedimentation assay (b) and microscopy (c) using EPYC1-GFP. Scale bar 15 µm. **d**,**e** Droplets can be labelled using cyanobacterial SeRubisco-GFP (scale bar 15 µm) (d), and exhibit fusion (scale bar 5 µm) (e). **f,** Representative droplet sedimentation assay used to generate part of the phase diagram shown in Fig. 1e. **g,** The effect of NaCl on droplet formation (30 µM Rubisco, 7.1/0.4 µM EPYC1/EPYC1-GFP). Scale bar 15 µm. **h,i** Phase separation requires both EPYC1 and Rubisco as assessed by droplet sedimentation (h) and microscopy (i). Scale bar 10 µm. **j**, Phase separation is robust up to 40 °C. **k**, EPYC1 (but not Rubisco) maintains solubility and the ability to phase separate with Rubisco after a 15 minute pre-incubation at 98 °C.

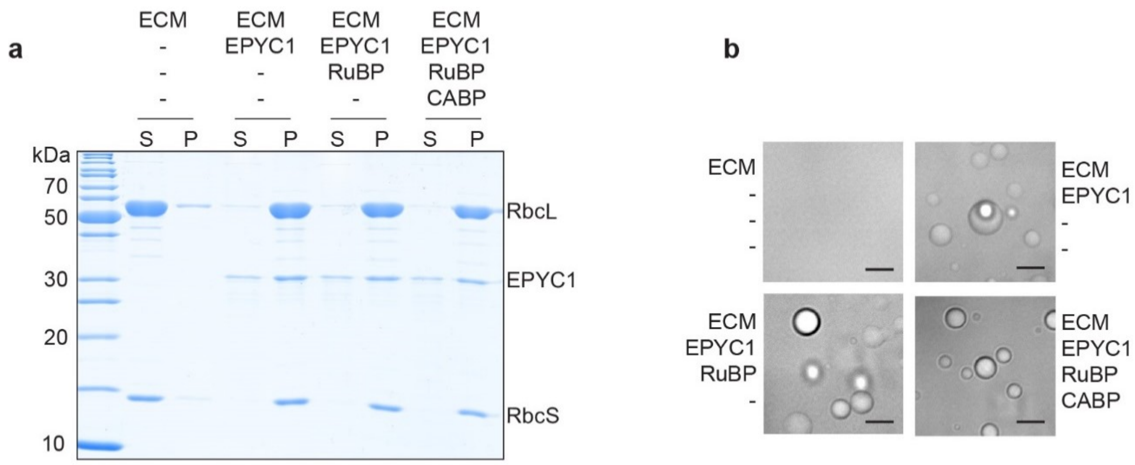

Supplementary Fig. 3| Quantitative Demixing of Rubisco during Two-Step Rubisco Activity Assay. Controls to Fig. 2b showing quantitative demixing of Rubisco and droplet formation via sedimentation assay (**a**) and visualization via microscopy (**b**), respectively. Scale bar 5 µm**.** Concentrations: ECM, 7.5 µM active sites; EPYC1, 3.75 µM; RuBP, 2.5 mM; CABP, 60 µM.

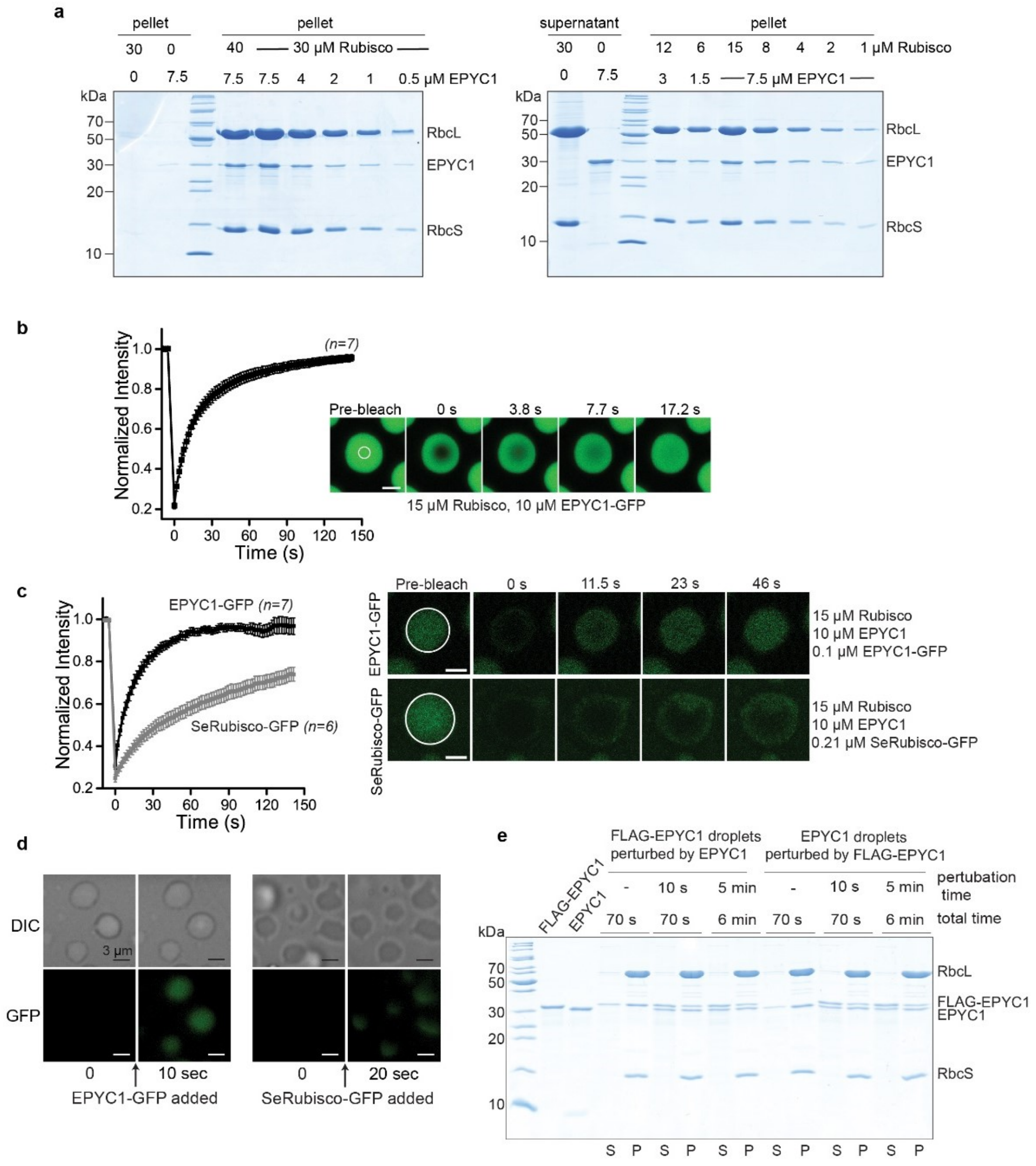

Supplementary Fig. 4| Characterization of droplet composition and component exchange. a, Droplet sedimentation assay performed while varying EPYC1 and Rubisco concentrations. One representative data set used to generate Figs. 3a and b. is shown. **b,** FRAP of droplets formed using Rubisco and EPYC1-GFP. Error bars indicate the s.e.m.. Scale bar 2 μm. **c,** Bleaching entire droplets suggests exchange of droplet and bulk solvent components occurs rapidly. Error bars indicate the s.e.m.. Scale bar 2 μm. **d,** EPYC1 (25 μM)-Rubisco (15 μM) droplets were allowed to form for three minutes prior to addition of 0.1 μM of EPYC1-GFP (left) or SeRubisco-GFP (right). Scale bar 3 μm. **e,** Component exchange assay similar to Fig. 3d, but following the exchange of EPYC1 and FLAG-EPYC1 using the droplet sedimentation assay. Rubisco (7.5 μM)-EPYC1 (3.75 μM) droplets were formed and perturbed using an equal quantity of FLAG-EPYC1 and incubated for 10 s or 5 minutes. Centrifugation and supernatant removal was performed within 60 s. Exchange was faster than can be resolved within the 70 s required for mixing and sedimentation.

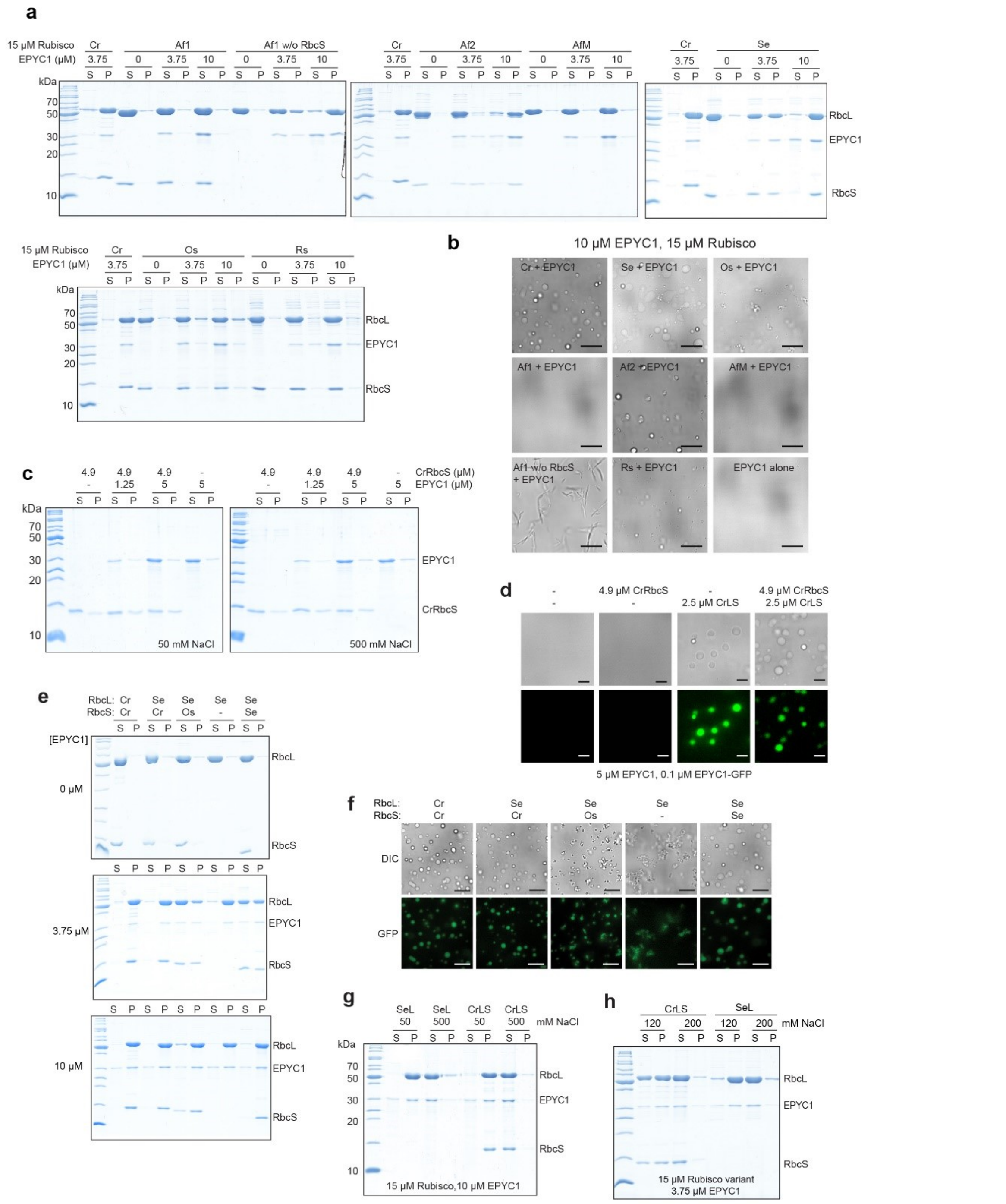

Supplementary Fig. 5| Demixing of EPYC1 with heterologous and chimeric Rubiscos. a,b Droplet sedimentation assay (a) and microscopy (b) of heterologous Rubiscos demixing with EPYC1. Data is the extended version of that shown in Figs. 4a and b. Scale bar 15 µm. **c,d** Sedimentation assay (c) and microscopy (d) of isolated Chlamydomonas Rubisco Small Subunit (CrRbcS) with EPYC1. Droplet formation necessitates inclusion of the Chlamydomonas holoenzyme (CrLS). Scale bar 5 µm. **e,** Droplet sedimentation assay of cyanobacterial and chimeric Rubiscos as shown abbreviated in Fig. 4c. **f,** Droplet morphology of cyanobacterial and chimeric demixed Rubiscos (extended version of Fig. 4d). Scale bar 15 µm. **g,** The formation of amorphous aggregates with cyanobacterial large subunits (SeL) is reversible by salt. Demixing took place first in buffer A and NaCl concentration was adjusted prior to centrifugation. **h,** SeL-droplets exhibit a similar salt-sensitivity to CrLS-droplets. NaCl concentration adjusted prior to protein addition.

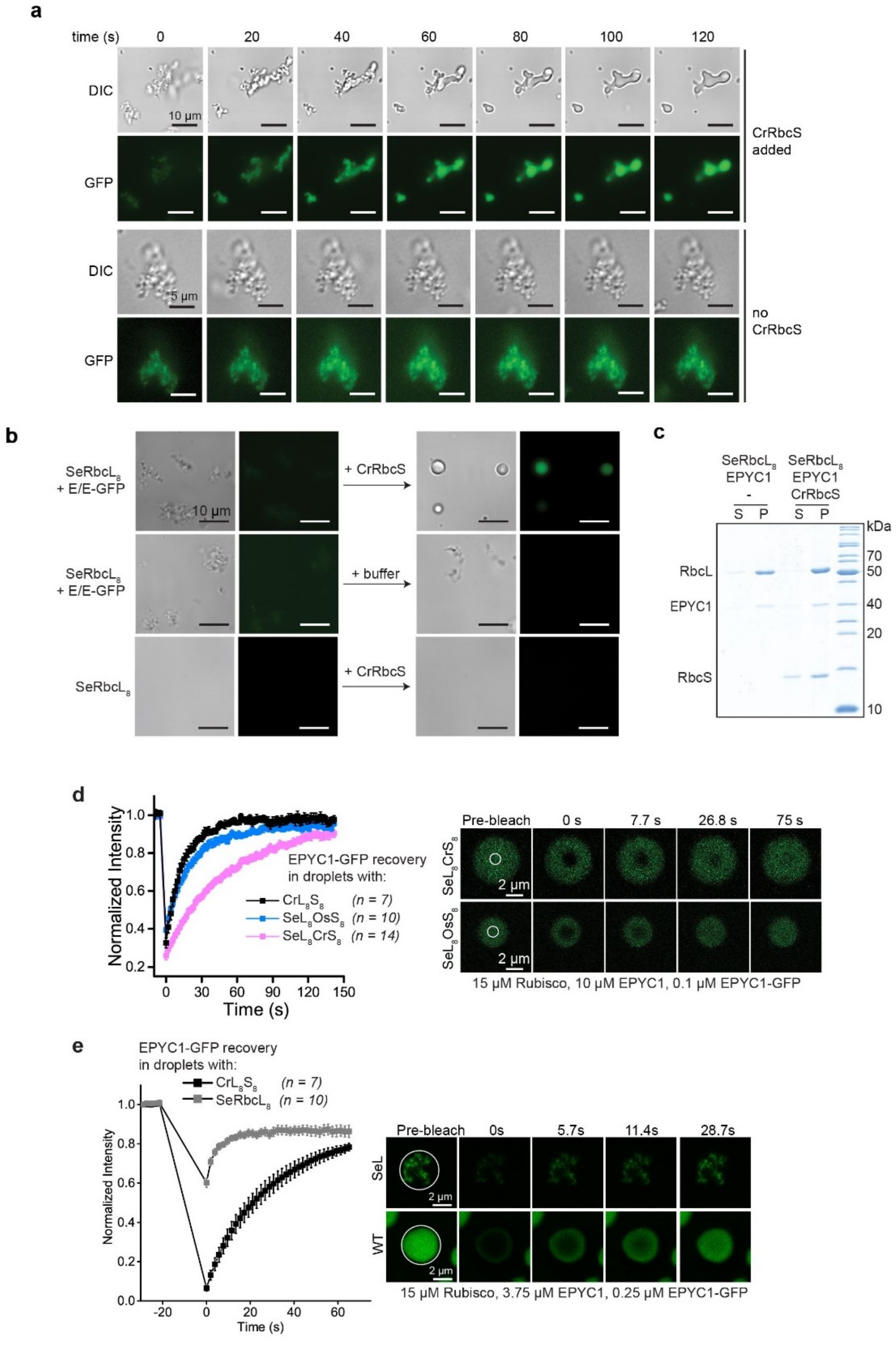

Supplementary Fig. 6| Control experiments concerning the transition of droplet morphology and analysis of droplets formed using non-native Rubiscos. a, Comparison of time lapse of the appearance of SeRbcL$_8$ (10 µM) -EPYC1/EPYC1-GFP (5/0.4 µM) droplets (2.5 µl) when CrRbcS (4 µM) or buffer A is added (10 µl final). Scale bar 10 µm (upper panel) or 5 µm (lower panel). **b,** Experiments equivalent to those shown in (a), but including the control that lacks EPYC1. Scale bar 10 µm. **c,** SDS-PAGE analysis of a droplet sedimentation assay prior and subsequent to CrRbcS addition confirms that the small subunit enters the droplets. **d,** FRAP assay of droplets formed with EPYC1/EPYC1-GFP using CrRubisco, and the chimeric Rubiscos SeL$_8$CrS$_8$ or SeL$_8$OsS$_8$. Error bars indicate s.e.m.. Scale bar 2 µm. **e,** FRAP assay of droplets formed with EPYC1/EPYC1-GFP using the Rubisco core SeL$_8$. Error bars indicated s.e.m.. Scale bar 2 µm.

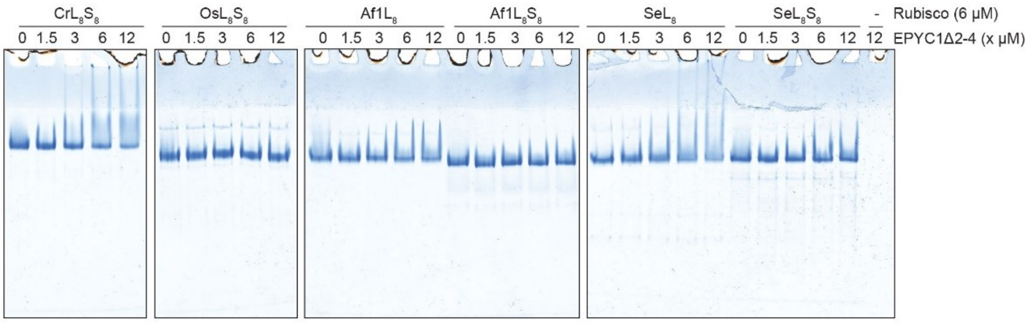

Supplementary Fig. 7| Native PAGE gelshift assay using Rubisco and the single repeat variant EPYC1Δ2-4. Full gels are shown for the experiment depicted in Fig. 4f.

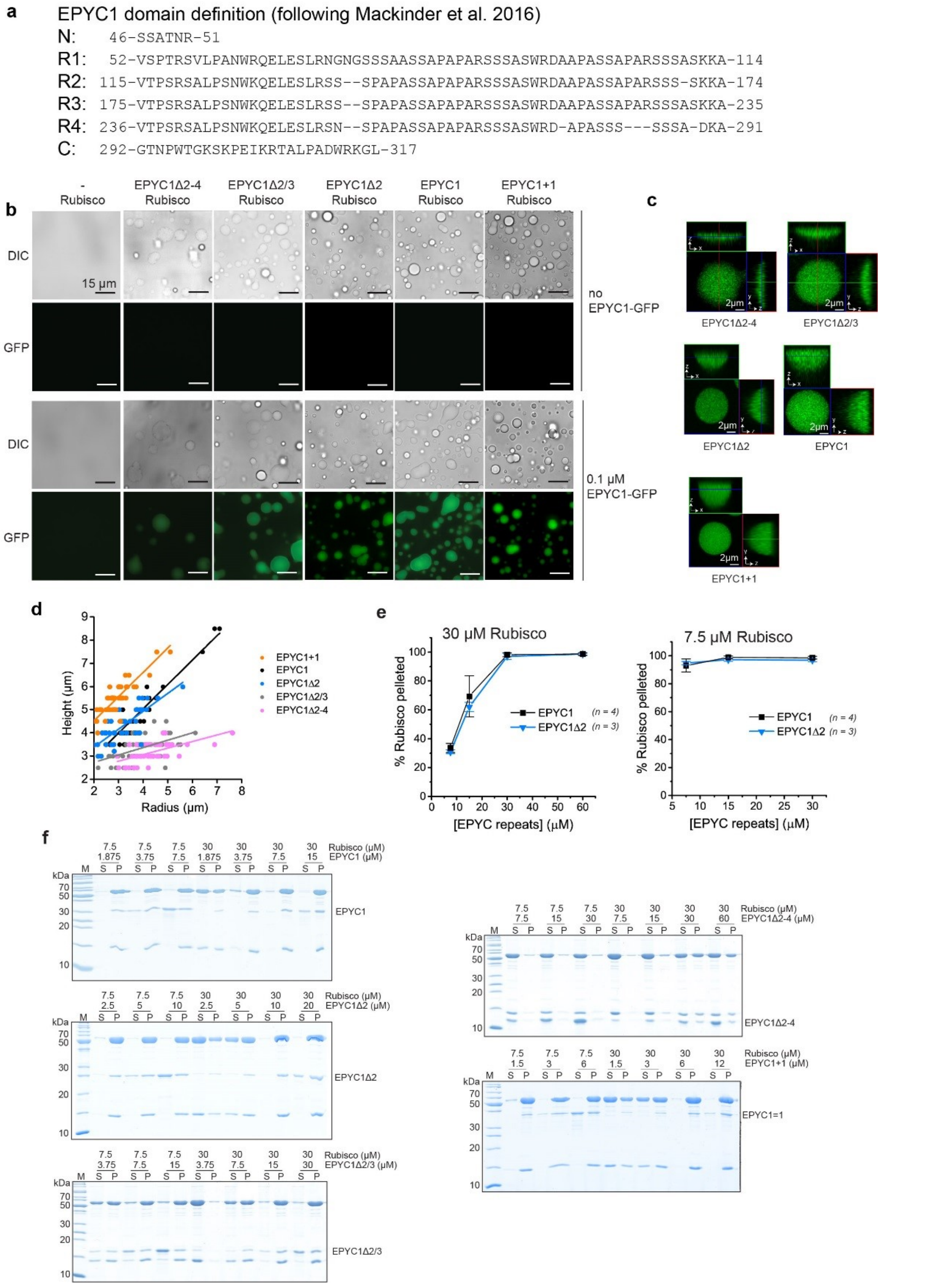

Supplementary Fig. 8| Characterization of droplets formed using EPYC1 fragments. a, Aminoacid sequences for the EPYC1 domains. **b,** Comparison of droplets (7.5 µM EPYC1 or repeat equivalent/ 30 µM Rubisco) formed in the presence and absence of labelling EPYC1-GFP. Scale bar 15 µm. **c,** z-stack analysis of representative droplets to Fig. 5c (conditions as for Fig. 5d). **d,** Distribution of droplet radius and height obtained from z-stack analysis. Scale bar 2 µm. **e,** Additional replicates of droplet sedimentation assay with wild-type EPYC1 and EPYC1Δ2 to address the theory of a 'magic number effect', see Fig. 5e. Error bars indicate s.d. **f,** SDS-PAGE analysis of droplet sedimentation assay used to generate Fig. 5e.

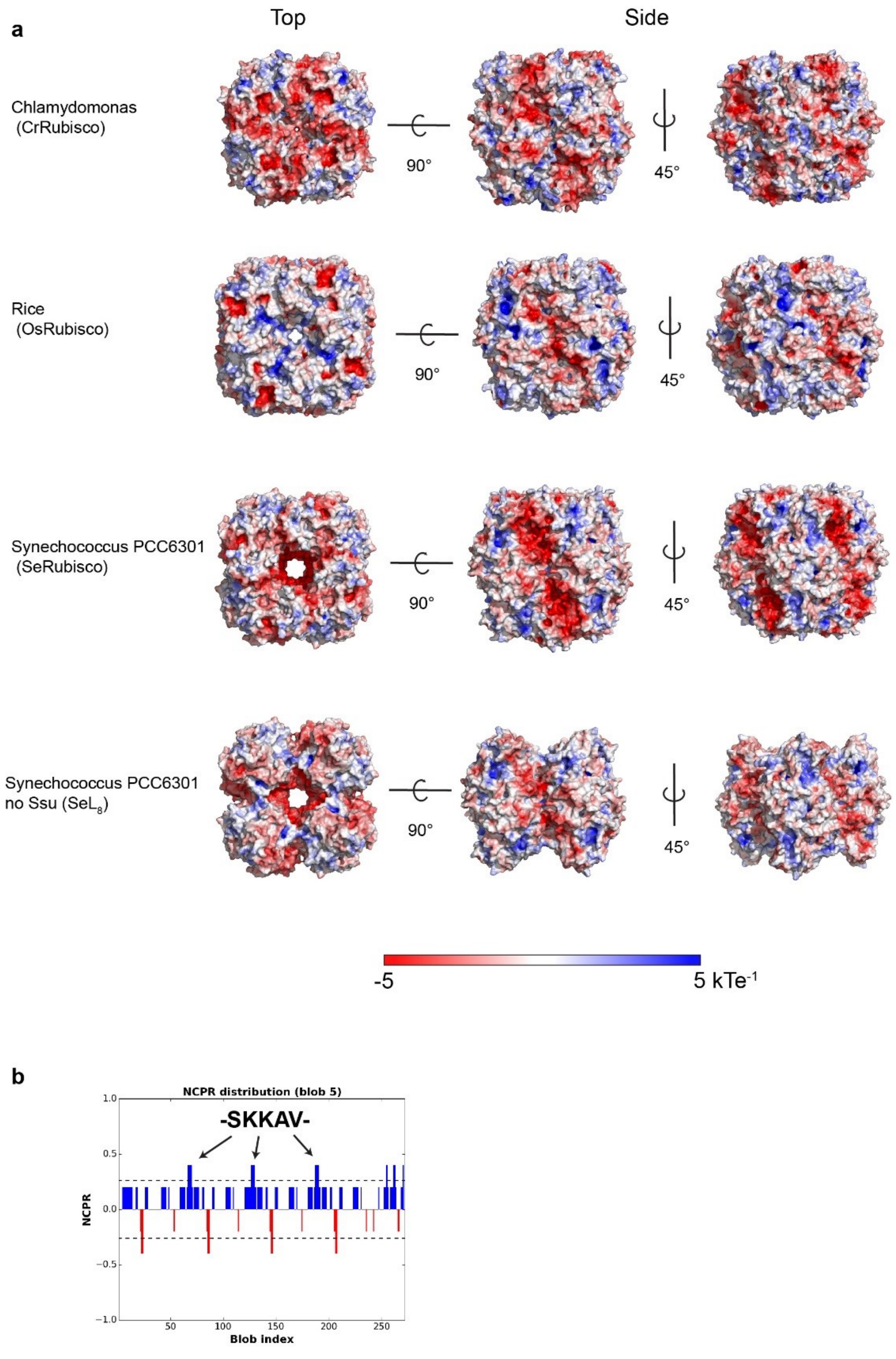

Supplementary Fig. 9| Electrostatic properties of Rubisco and EPYC1. **a,** Surface charge of available crystal structures was calculated using the APBS plugin[1] in PYMOL (www.pymol.org). The potential on the solvent accessible surface was coloured in the indicated range. CrRubisco, PDB:1GK8; OsRubisco, PDB:1WDD; SeRubisco/SeL_8, PDB:1RBL. **b,** EPYC1 NPCR (net charge per residue) was calculated for five residue stretches (blobs) using the CIDER webtool[2]. Three positively charged blobs correspond to the SKKAV motif as indicated.

Supplementary Table 1| Plasmids used in this study

Plasmid	Encoded gene product	Reference
pHue*CrEPYC1*	His_6-Ub-CrEPYC1 (UniProt A0A2K3DA85, residues 46-317)	This work
pHue*FLAGCrEPYC1*	His_6-Ub-FLAG-CrEPYC1	This work
pHue*FLAGCrEPYC1GFP*	His_6-Ub-FLAG-CrEPYC1-GFP*	This work
pHue*CrEPYC1Δ2*	His_6-Ub-CrEPYC1Δ2*	This work
pHue*CrEPYC1Δ2/3*	His_6-Ub-CrEPYC1Δ2/3*	This work
pHue*CrEPYC1Δ2-4*	His_6-Ub-CrEPYC1Δ2-4*	This work
pHue*CrEPYC1+1*	His_6-Ub-CrEPYC1+1*	This work
pTrc*SynLF345IS*	*Synechococcus* PCC6301 (Se) RbcL (F345I) and RbcS	Mueller-Cajar and Whitney 2008[3]
pTrc*SynLF345I*	*Synechococcus* PCC6301 RbcL (F345I)	This work
pTrc*SynLF345IS-GFP*	*Synechococcus* PCC6301 RbcL (F345I) and RbcS-GFP*	This work
pET22b*CrRbcS*	CrRbcS (UniProt P00873, residues 46-185)	This work
pET22b*OsRbcS*	*Oryza sativa* subsp. *japonica* OsRbcS (UniProt P18566, residues 48-175)	This work
pET30b*AfcbbLS*	*Acidithiobacillus ferrooxidans* AfCbbL and AfCbbS	Tsai et al. 2015[4]
pHue*AfcbbL*	His_6-Ub-AfCbbL *Acidithiobacillus ferrooxidans* AfCbbL	This work
pHue*AfcbbM*	*A. ferrooxidans* AfM	Tsai et al. 2015
pET24b*Af2cbbLS*	*A. ferrooxidans* UniProt Af2CbbL (UniProt B7JB30) and Af2CbbS (UniProt B7JB29)	This work
pET30b*RscbbLS*	*Rhodobacter sphaeroides* (Rs) RbcL and RbcS	Mueller-Cajar et al. 2011[5]

* amino acid sequence given in Supplementary Table 2

Supplementary Table 2| Sequences of selected proteins

Protein	Amino acid sequence
FLAG-CrEPYC1-GFP (EPYC1-GFP)	*DYKDDDDK*SSATNRVSPTRSVLPANWRQELESLRNGNGSSSAASSAPAPARSSSASWRDAAPASSAPARSSSASKKAVTPSRSALPSNWKQELESLRSSSPAPASSAPAPARSSSASWRDAAPASSAPARSSSSKKAVTPSRSALPSNWKQELESLRSSSPAPASSAPAPARSSSASWRDAAPASSAPARSSSASKKAVTPSRSALPSNWKQELESLRSNSPAPASSAPAPARSSSASWRDAPASSSSSSADKAGTNPWTGKSKPEIKRTALPADWRKGLGSLGGSGGRMVSKGEELFTGVVPILVELDGDVNGHKFSVRGEGEGDATNGKLTLKFICTTGKLPVPWPTLVTTLTYGVQCFSRYPDHMKQHDFFKSAMPEGYVQERTISFKDDGTYKTRAEVKFEGDTLVNRIELKGIDFKEDGNILGHKLEYNFNSHNVYITADKQKNGIKANFKIRHNVEDGSVQLADHYQQNTPIGDGPVLLPDNHYLSTQSKLSKDPNEKRDHMVLLEFVTAAGITLGMDELYK*
CrEPYC1Δ2	SSATNRVSPTRSVLPANWRQELESLRNGNGSSSAASSAPAPARSSSASWRDAAPASSAPARSSSASKKAVTPSRSALPSNWKQELESLRSSSPAPASSAPAPARSSSASWRDAAPASSAPARSSSASKKAVTPSRSALPSNWKQELESLRSNSPAPASSAPAPARSSSASWRDAPASSSSSSADKAGTNPWTGKSKPEIKRTALPADWRKGL
CrEPYC1Δ2/3	SSATNRVSPTRSVLPANWRQELESLRNGNGSSSAASSAPAPARSSSASWRDAAPASSAPARSSSASKKAVTPSRSALPSNWKQELESLRSNSPAPASSAPAPARSSSASWRDAPASSSSSSADKAGTNPWTGKSKPEIKRTALPADWRKGL
CrEPYC1Δ2-4	SSATNRVSPTRSVLPANWRQELESLRNGNGSSSAASSAPAPARSSSASWRDAAPASSAPARSSSASKKAGTNPWTGKSKPEIKRTALPADWRKGL
CrEPYC1+1	SSATNRVSPTRSVLPSNWKQELESLRSSSPAPASSAPAPARSSSASWRDAAPASSAPARSSSASKKAVTPSRSALPSNWKQELESLRSSSPAPASSAPAPARSSSASWRDAAPASSAPARSSSSKKAVTPSRSALPSNWKQELESLRSSSPAPASSAPAPARSSSASWRDAAPASSAPARSSSSKKAVTPSRSALPSNWKQELESLRSSSPAPASSAPAPARSSSASWRDAAPASSAPARSSSASKKAVTPSRSALPSNWKQELESLRSNSPAPASSAPAPARSSSASWRDAPASSSSSSADKAGTNPWTGKSKPEIKRTALPADWRKGL
SeRbcS-GFP	MSMKTLPKERRFETFSYLPPLSDRQIAAQIEYMIEQGFHPLIEFNEHSNPEEFYWTMWKLPLFDCKSPQQVLDEVRECRSEYGDCYIRVAGFDNIKQCQTVSFIVHRPGRYGSNGDLGGSGGRVSKGEELFTGVVPILVELDGDVNGHKFSVRGEGEGDATNGKLTLKFICTTGKLPVPWPTLVTTLTYGVQCFSRYPDHMKQHDFFKSAMPEGYVQERTISFKDDGTYKTRAEVKFEGDTLVNRIELKGIDFKEDGNILGHKLEYNFNSHNVYITADKQKNGIKANFKIRHNVEDGSVQLADHYQQNTPIGDGPVLLPDNHYLSTQSKLSKDPNEKRDHMVLLEFVTAAGITLGMDELYK*

*sequence of FLAG-tag in *italics*, linker sequence between protein of interest and GFP-tag underlined

Supplementary References

1 Baker, N. A., Sept, D., Joseph, S., Holst, M. J. & McCammon, J. A. Electrostatics of nanosystems: application to microtubules and the ribosome. *Proc. Natl. Acad. Sci. U. S. A.* **98**, 10037-10041, doi:10.1073/pnas.181342398 (2001).

2 Holehouse, A. S., Das, R. K., Ahad, J. N., Richardson, M. O. & Pappu, R. V. CIDER: Resources to Analyze Sequence-Ensemble Relationships of Intrinsically Disordered Proteins. *Biophysical Journal* **112**, 16-21, doi:10.1016/j.bpj.2016.11.3200 (2017).

3 Mueller-Cajar, O. & Whitney, S. M. Evolving improved Synechococcus Rubisco functional expression in Escherichia coli. *Biochem J* **414**, 205-214 (2008).

4 Tsai, Y. C., Lapina, M. C., Bhushan, S. & Mueller-Cajar, O. Identification and characterization of multiple rubisco activases in chemoautotrophic bacteria. *Nat Commun* **6**, 8883, doi:10.1038/ncomms9883 (2015).

5 Mueller-Cajar, O. *et al.* Structure and function of the AAA+ protein CbbX, a red-type Rubisco activase. *Nature* **479**, 194-199 (2011).
