# Supplementary material for: The phase separation underlying the pyrenoid-based microalgal Rubisco supercharger
Source: Nat Commun. 2018 Nov 29;9:5076. doi: 10.1038/s41467-018-07624-w (PMC6265248; doi:10.1038/s41467-018-07624-w)
Supplement: Supplementary file 3 — Description of Additional Supplementary Files [file 41467_2018_7624_MOESM3_ESM.pdf]

Description of Additional Supplementary Files

File Name: Supplementary Movie 1
Description: The EPYC1-Rubisco phase separation
Droplets were generated using 15 µM Rubisco and 10 µM EPYC1 spiked with 0.1 µM EPYC1-GFP. After 3 min, DIC and epifluorescence images were acquired every 2 s, channels were pseudocolored and merged in ImageJ/FiJi, and exported with 10 frames per second, resulting in a 20x accelerated movie.

File Name: Supplementary Movie 2
Description: Transition of droplet morphology upon small subunit addition
Droplets were generated as described for Fig. 4e. 10 s after addition of CrRbcS, DIC and epifluorescence images were acquired every 2 s, channels were pseudocolored and merged in ImageJ/FiJi, and exported with 10 frames per second, resulting in a 20x accelerated movie.
